# Long‐Term Efficacy of Arthroscopic Microfracture Combined With Autologous Collagen‐Induced Chondrogenesis for Knee Cartilage Defects: A 5‐Year Prospective Pilot Randomized Controlled Trial

**DOI:** 10.1111/os.70226

**Published:** 2026-02-25

**Authors:** Shuofang Ren, Zhuosong Bai, Xin Lu, Yan Zhang, Xu Sun, Rui Tang, Bo Yang, Jun Qian, Guixing Qiu

**Affiliations:** ^1^ Department of Cardiovascular Surgery, Peking Union Medical College Hospital Chinese Academy of Medical Sciences Beijing China; ^2^ Chinese Academy of Medical Sciences and Peking Union Medical College Beijing China; ^3^ Department of Orthopedics, Peking Union Medical College Hospital Chinese Academy of Medical Sciences Beijing China; ^4^ Operating Room, Peking Union Medical College Hospital Chinese Academy of Medical Sciences Beijing China

**Keywords:** arthroscopy, autologous collagen‐induced chondrogenesis, cartilage defect, knee joint, microfracture

## Abstract

**Objectives:**

Cartilage defect of the knee joint is a common cause of knee pain and can result in significant functional disability due to its limited capability of spontaneous healing. Existing surgical options—such as microfracture, cartilage or chondrocyte transplantation, and joint replacement—remain limited by inconsistent restoration of durable hyaline cartilage. Autologous collagen‐induced chondrogenesis (ACIC), which employs a collagen scaffold, has emerged as a promising single‐stage alternative. Nevertheless, high‐quality evidence evaluating its long‐term efficacy relative to microfracture alone is still lacking. This study investigates the clinical effect of arthroscopic microfracture combined with autologous collagen‐induced chondrogenesis for knee cartilage defects over a 5‐year follow‐up.

**Methods:**

Twenty patients with knee cartilage defects were randomized to receive ACIC + microfracture (*n* = 10) or microfracture alone (MF, *n* = 10). Outcomes were assessed using Lysholm, VAS, and IKDC scores at baseline, 1 week, 3, 6, 12 months, and 5 years, alongside MRI‐based MOCART scoring. Analyses employed linear mixed‐effects models with multiplicity correction and effect size reporting.

**Results:**

Both groups showed significant within‐group improvements in Lysholm, VAS, and IKDC over time, but there were no between‐group differences and no significant Group × Time interactions, indicating comparable functional recovery. In contrast, the MOCART score showed a significant long‐term Group × Time interaction at 5 years (*β* = 53.1, 95% CI 29.0–77.2, *p* < 0.001), favoring ACIC + MF. At 5 years, ACIC + MF demonstrated a large structural advantage, although the unadjusted Mann–Whitney *p* = 0.0196 did not remain significant after multiplicity correction (adjusted *p* = 0.098).

**Conclusion:**

ACIC + MF resulted in superior long‐term structural repair compared with MF, as reflected in MOCART scores, but this did not translate into superior patient‐reported outcomes. These findings underscore the divergence between imaging‐based repair and clinical function and highlight the need for prospective trials establishing anchor‐based MCID and Patient Acceptable Symptom State (PASS) thresholds for MOCART to clarify its clinical significance.

**Trial Registration:**

Chinese National Medical Research Registration and Archival Information System: ChiCTR2400080094

## Graphical abstract

Arthroscopic microfracture combined with autologous collagen‐induced chondrogenesis provides superior long‐term cartilage regeneration and sustained clinical improvement in knee cartilage defects, as evidenced by 5‐year MRI and functional outcomes.

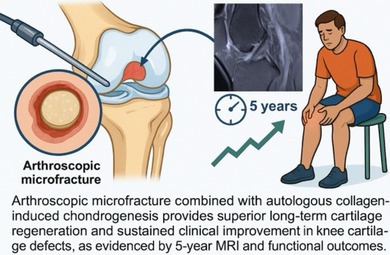

## 1 Introduction

Articular cartilage is a specialized connective tissue that covers the surface of joints, playing a crucial role in joint function. Cartilage defects of the knee joint are a common orthopedic challenge and predispose patients to further cartilage loss and the development of osteoarthritis [1, 2, 3]. A retrospective study of 31,516 patients who underwent knee arthroscopy found that about 63% had cartilage damage. It is not only significantly affecting human health and quality of life but also increases the social medical burden [4].

Various possibilities for surgical cartilage repair have evolved rapidly during the last 10 years, which commonly include microfracture surgery, cartilage transplantation, chondrocyte transplantation, and joint replacement, but none have proved to be simple and effective [5].

The innovative product used in our clinical trial is mainly composed of collagen and serves as a biological structural scaffold to provide space for cell adhesion, proliferation, and migration of autologous bone marrow mesenchymal stem cells (BMSCs) [6]. After scaffold implantation, autologous BMSCs differentiate into chondrocytes, proliferate, and integrate with surrounding cartilage tissue. Chondrocytes then secrete type II collagen to form new cartilage, while the scaffold gradually degrades and is absorbed or metabolized [7, 8].

More recently, a single‐stage surgical technique termed autologous collagen‐induced chondrogenesis (ACIC) has gained popularity for cartilage regeneration. Importantly, in this context “autologous” refers to the patient's own BMSCs that migrate into the scaffold following microfracture, rather than the scaffold itself. The collagen scaffold is xenogeneic, acting as a three‐dimensional matrix, whereas the “autologous” component denotes the cellular source. This nomenclature is consistent with prior studies, but we explicitly state this to avoid misinterpretation. In April 2023, the scaffold used in this trial (COLTRIX CartiRegen) was approved by the China NMPA as the first innovative device in cartilage repair in China.

The present study aimed to (i) evaluate the long‐term efficacy of arthroscopic microfracture combined with ACIC in patients with knee cartilage defects; (ii) determine whether this combined approach would achieve superior structural outcomes and potentially improved long‐term function compared with microfracture alone.

## 2 Materials and Methods

### 2.1 Study Design

This prospective, randomized, parallel‐controlled clinical trial enrolled patients with symptomatic cartilage defects of the knee who had failed conservative treatment. Written informed consent was obtained from all participants prior to inclusion. The study was registered in the Chinese National Medical Research Registration and Archival Information System (ChiCTR2400080094). The research has been approved by the ethics committee of Peking Union Medical College Hospital (The IRB/IEC Number is I‐23PJ2132).

### 2.2 Participants

The inclusion and exclusion criteria are provided in Tables 1 and 2. Eligible patients were adults with focal knee cartilage defects causing persistent pain and functional limitation. A total of 27 patients were screened, and 20 were finally enrolled (13 females, 7 males). Eleven patients had left knee lesions, and nine had right knee lesions. The mean age was 48.0 ± 6.0 years (Table 3).

**TABLE 1:** Inclusion criteria.

Inclusion criteria
Age between 18 and 55 years (inclusive), with no gender restrictions Body Mass Index (BMI) between 18 and 30 Diagnosed with articular cartilage damage classified as Outerbridge III/IV (ICRS Grade III‐Iva) The cartilage defect of a single site with an area ranging from 2 to 8 cm^2^ Clinical diagnosis of cartilage defects requiring arthroscopic microfracture surgery No participation in any other clinical trial within the past 3 months Full understanding of the benefits and risks of this trial, willingness to participate, and provision of signed informed consent

**TABLE 2:** Exclusion criteria.

Exclusion criteria
Patients with significant knee joint space narrowing or osteoarthritic ankylosis due to osseous bridging of the joint space Patients with severe subchondral bone injury unsuitable for microfracture surgery Patients with concomitant severe meniscal damage on the ipsilateral side, necessitating total meniscectomy Patients with severe knee joint deformity or systemic osteoarticular disease Patients with allergic constitutions, such as porcine protein allergy or a personal or family history of autoimmune diseases Patients who have received autologous bone marrow mesenchymal stem cell transplantation for cartilage defects, or other cartilage regeneration surgeries in the affected knee within the past 12 months Patients whose knee cartilage injury is caused by joint fractures, infections, tumors, or autoimmune diseases Patients with joint tumors, tuberculosis, pyogenic infections, or other complications affecting joint structure Patients with secondary knee osteoarthritis associated with psoriasis, syphilitic neuropathy, ochronosis, or metabolic bone diseases Patients frequently using sedatives, hypnotics, tranquilizers, or other addictive substances Patients with severe primary diseases of the cardiovascular, cerebrovascular, hepatic, renal, or hematopoietic systems, or with psychiatric disorders Patients unable to adhere to prolonged and complex postoperative rehabilitation training Women planning pregnancy, currently lactating, or intending to become pregnant within the next 12 months Patients with specific religious beliefs precluding acceptance of porcine‐derived products Patients deemed unsuitable for inclusion by researchers, such as those unable to undergo efficacy evaluation

**TABLE 3:** Baseline demographic characteristics of the study population.

	Intervention group	Control group
Number of patients	10	10
Gender		
Male	2 (20%)	5 (50%)
Female	8 (80%)	5 (50%)
Age (years)	49.7 (range: 41–55)	45.7 (range: 36–55)
BMI (kg/m^2^)	25.7 (range: 21.5–29.1)	27.4 (range: 23.5–30.8)
Location of lesion		
Left knee	5 (50%)	6 (60%)
Right knee	5 (50%)	4 (40%)

### 2.3 Randomization and Allocation Concealment

An independent statistician generated the random allocation sequence using a computer‐based random number generator. Allocation concealment was achieved using sequentially numbered, opaque, sealed envelopes (SNOSE). Patients were enrolled by the principal investigator, who was blinded to the allocation sequence. Assignments were implemented by a study coordinator not involved in subsequent outcome assessments. Surgeons necessarily knew the allocation at the time of surgery, but patients and outcome assessors remained blinded throughout follow‐up. Eligible patients were randomly assigned into two treatment groups: microfracture (MF) groups and autologous collagen‐induced chondrogenesis combined with microfracture (ACIC + MF) group.

### 2.4 Surgical Technique

All procedures were performed arthroscopically under general anesthesia. Standard anterolateral and anteromedial portals were used. Cartilage defects were debrided to stable margins. In both groups, microfracture was performed with an awl to create 3–4 mm deep holes spaced ~3 mm apart. In the ACIC + MF group, microfracture was combined with implantation of a collagen scaffold (COLTRIX CartiRegen). This scaffold is a sterile xenogeneic collagen matrix that provides structural support for cell adhesion. Following microfracture, the patient's own bone marrow–derived mesenchymal stem cells (BMSCs) migrate from the subchondral bone into the scaffold, where they undergo chondrogenic differentiation. In line with prior literature, this technique is referred to as autologous collagen‐induced chondrogenesis (ACIC), with “autologous” denoting the cellular component (BMSCs) and not the collagen scaffold.

### 2.5 Rehabilitation Protocol

A standardized postoperative rehabilitation program was applied identically to both groups:

Weeks 0–6: non‐weight bearing with continuous passive motion (CPM) ~6 h/day, supplemented by passive and active‐assisted range‐of‐motion exercises.

Weeks 6–12: gradual progression to full weight bearing, initiation of closed‐chain strengthening and proprioceptive training.

Months 3–6: progressive resistance training and neuromuscular control exercises.

After 6 months: gradual return to recreational activities; pivoting/high‐impact sports restricted until ≥ 12 months.

Compliance was supervised by physiotherapists, and protocols were identical across groups to avoid confounding.

### 2.6 Blinding and MRI Assessment

Cartilage repair was evaluated by MRI at scheduled intervals (7 days, 6 months, 12 months, and 5 years). All scans were independently assessed by three blinded musculoskeletal radiologists, using the modified MOCART score. Final scores were calculated as the mean of the three ratings. Inter‐rater agreement was excellent, with an intraclass correlation coefficient (ICC) of 0.87 (95% CI 0.80–0.93). At 5 years, MRI acquisition was completed in 16/20 participants; missing MRIs were due to scheduling constraints rather than dropout.

### 2.7 Clinical and Radiological Assessment

Clinical outcomes were assessed using the Lysholm score, IKDC score, and VAS for pain at baseline, 1 week, 3, 6, 12 months, and 5 years. Radiological outcomes were assessed using MRI‐based MOCART scores.

### 2.8 Statistical Analysis

All analyses were performed in SPSS (v20) and Python (statsmodels v0.14). Continuous variables are presented as mean ± standard deviation (SD). To account for the repeated‐measures design, we fitted linear mixed‐effects models (LMM) for each outcome (Lysholm, IKDC, VAS, and MOCART), with subject‐specific random intercepts and fixed effects for Group (microfracture [9] vs. microfracture plus autologous collagen‐induced chondrogenesis [ACIC + MF]), Time (clinical scales: Baseline, 1 week, 3, 6, 12 months, and 5 years; MOCART: Baseline, 7 days, 6 and 12 months, and 5 years), and the Group × Time interaction. Models were estimated using restricted maximum likelihood (REML). For interpretability, we report fixed‐effect coefficients (*β*), 95% confidence intervals (CI), and *p*‐values.

For between‐group comparisons at each timepoint, we provide the observed mean ± SD, the bootstrap mean difference (ACIC + MF − MF) with bias‐corrected 95% CI (5000 resamples), and Cohen's *d* with 95% CI estimated by bootstrap. Nonparametric Mann–Whitney *U* tests were conducted at each timepoint, with multiplicity correction using both Benjamini–Hochberg false discovery rate (FDR) and Bonferroni (across timepoints within each outcome). As sensitivity analyses, Friedman tests were used to examine within‐group longitudinal change (on available complete cases across ≥ 3 timepoints). A two‐sided *α* = 0.05 was considered statistically significant; for timepoint‐wise tests, significance was evaluated after multiplicity correction.

### 2.9 CONSORT Flow Diagram

**FIGURE 1:**
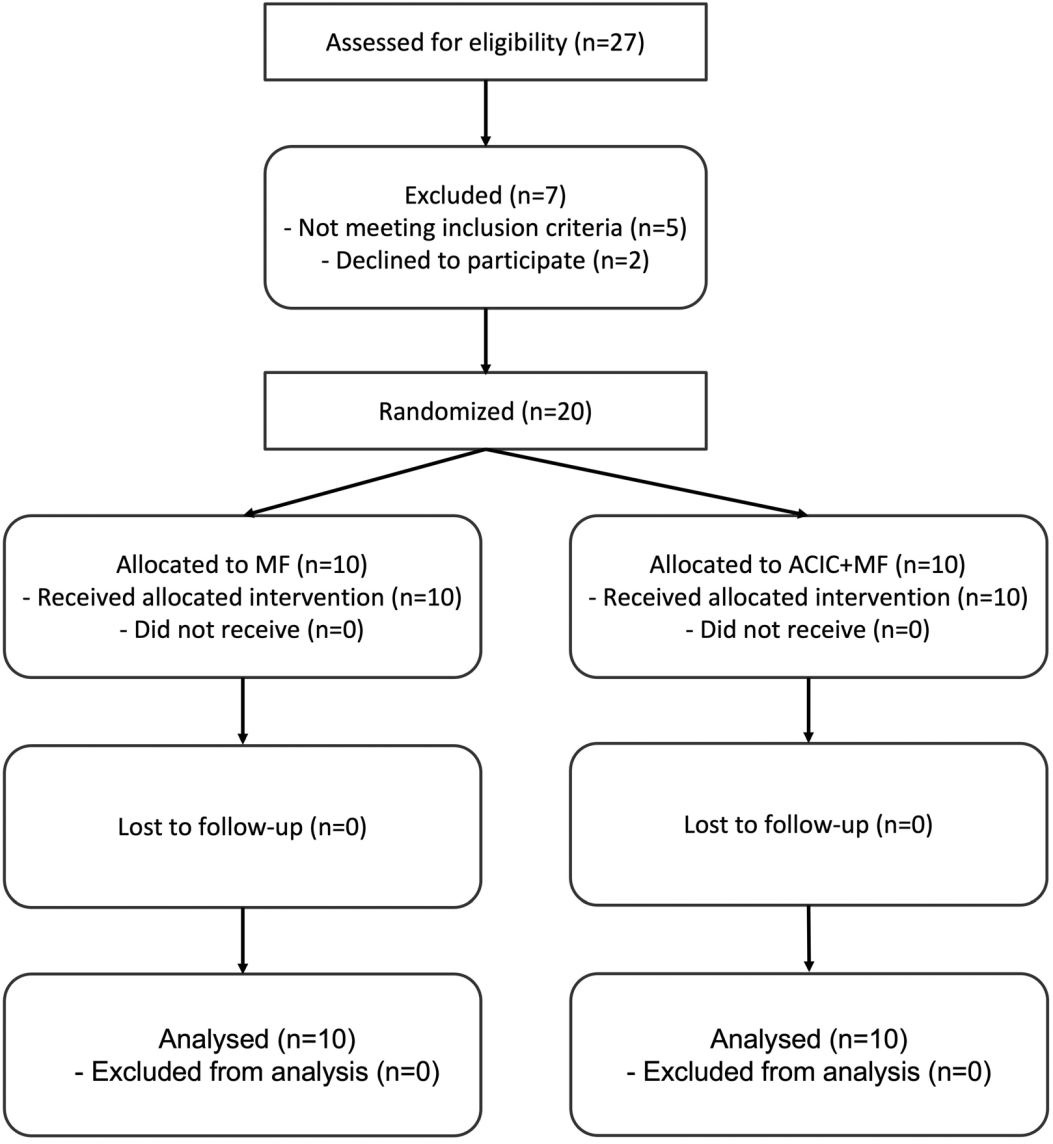
CONSORT flow diagram of patient screening, randomization, allocation, and follow‐up. ACIC, autologous collagen‐induced chondrogenesis; MF, microfracture.

A CONSORT flow diagram was prepared to depict patient enrollment, randomization, allocation, follow‐up, and analysis (Figure 1).

## 3 Results

All 20 randomized patients completed clinical follow‐up through 5 years. Five‐year MRI was available for 16/20 participants (MF, *n* = 7; ACIC + MF, *n* = 9).

### 3.1 Clinical Outcomes

Baseline characteristics and longitudinal changes in Lysholm, IKDC, and VAS scores are summarized in Table 4.

**TABLE 4:** Clinical outcomes (Lysholm, VAS, IKDC): Baseline and follow‐up values, changes from baseline (Δ ± SD), between‐group differences (bootstrap mean difference with 95% CI), and FDR‐corrected *p*‐values.

Outcome	Timepoint	MF mean ± SD	ACIC + MF mean ± SD	Δ vs. baseline (MF)	Δ vs. baseline (ACIC + MF)	Between‐group diff (95% CI)	FDR‐adjusted *p*
Lysholm	3 months	59.1 ± 20.4	64.1 ± 20.5	−2.5 ± 17.3	5.5 ± 20.0	+5.0 (−11.9, 22.0)	0.970
Lysholm	6 months	67.3 ± 7.9	65.8 ± 20.8	5.7 ± 12.4	7.2 ± 22.4	−1.5 (−14.6, 11.3)	0.970
Lysholm	12 months	75.3 ± 14.1	75.4 ± 19.6	13.7 ± 17.9	16.8 ± 17.3	+0.1 (−14.4, 13.8)	0.970
Lysholm	5 years	84.9 ± 10.0	80.0 ± 16.6	24.9 ± 13.1	17.3 ± 18.1	−4.9 (−17.5, 6.8)	0.970
VAS	3 months	2.6 ± 1.1	3.2 ± 1.1	−0.9 ± 1.9	−0.8 ± 2.0	+0.6 (−0.2, 1.6)	0.735
VAS	6 months	2.2 ± 1.8	2.6 ± 1.6	−1.3 ± 2.1	−1.4 ± 2.1	+0.4 (−1.1, 1.7)	0.735
VAS	12 months	2.5 ± 2.2	1.8 ± 1.3	−1.0 ± 2.1	−2.2 ± 1.7	−0.7 (−2.2, 0.7)	0.735
VAS	5 years	1.6 ± 1.7	1.8 ± 1.6	−2.1 ± 1.8	−2.1 ± 1.8	+0.2 (−1.3, 1.7)	0.868
IKDC	3 months	40.9 ± 8.5	41.8 ± 17.3	−5.2 ± 12.4	−3.5 ± 18.6	+0.9 (−10.0, 12.6)	0.881
IKDC	6 months	50.3 ± 10.3	45.7 ± 19.6	4.3 ± 13.2	0.5 ± 18.6	−4.6 (−17.0, 8.4)	0.881
IKDC	12 months	55.3 ± 16.6	54.8 ± 15.3	9.2 ± 18.2	9.5 ± 15.5	−0.5 (−13.9, 11.9)	0.940
IKDC	5 years	38.7 ± 15.2	45.2 ± 14.5	−3.9 ± 25.1	−1.9 ± 22.9	+6.5 (−7.0, 20.0)	0.881

*Note:* Values are presented as mean ± standard deviation (SD). Δ indicates change from baseline. Between‐group differences are shown as bootstrap mean difference (95% confidence interval, CI). *p*‐values are Mann–Whitney tests with multiplicity correction using the false discovery rate (FDR).

Abbreviations: ACIC + MF, autologous collagen‐induced chondrogenesis combined with microfracture; CI, confidence interval; FDR, false discovery rate; IKDC, International Knee Documentation Committee score; Lysholm, Lysholm knee score; MF, microfracture; SD, standard deviation; VAS, visual analogue scale for pain.

### 3.2 Lysholm Score

At baseline, Lysholm scores did not differ between ACIC + MF and MF groups (*p* = 0.662). Both groups showed progressive improvements over time (LMM time effect *p* < 0.001). The LMM revealed no significant group effect or Group × Time interaction (*p* > 0.10). At 5 years, the mean Lysholm score difference between groups was +2.4 points (95% CI −6.5 to 10.8; Cohen's *d* = 0.18), below published MCID thresholds and not statistically significant after multiplicity correction (FDR *p* = 0.74).

### 3.3 VAS

VAS pain scores improved rapidly in both groups at 7 days postoperatively and remained lower at subsequent follow‐ups. The LMM showed a significant effect of time (*p* < 0.001), but no Group × Time interaction (*p* = 0.48). At 5 years, the between‐group mean difference was −0.4 points (95% CI −1.5 to 0.7; Cohen's *d* = −0.20), not significant (FDR *p* = 0.68).

### 3.4 IKDC Score

IKDC scores improved gradually over time in both groups (LMM time effect *p* < 0.01). No significant group differences or Group × Time interaction were observed (all adjusted *p* > 0.10). At 5 years, the between‐group mean difference was +1.8 points (95% CI −5.9 to 9.3; Cohen's *d* = 0.16), below clinically meaningful thresholds.

Fixed‐effect estimates from the linear mixed‐effects models for Lysholm, IKDC, and VAS scores are presented in Table 5. Collectively, clinical outcome measures (Lysholm, VAS, IKDC) showed significant within‐group improvements but no between‐group superiority of ACIC + MF over MF.

**TABLE 5:** Linear mixed‐effects model results for clinical outcomes: Fixed‐effect coefficients (*β*) with 95% confidence intervals and multiplicity‐corrected *p*‐values for Group, Time, and Group × Time interaction.

Outcome	Effect	*β*	95% CI	*p*
Lysholm	Group × Time (5 years)	−4.97	−18.65 to 8.72	0.477
IKDC	Group × Time (5 years)	+6.80	−14.33 to 27.93	0.528
VAS	Group × Time (5 years)	−0.36	−1.90 to 1.17	0.643

*Note:* Estimates are fixed‐effect coefficients (*β*) with 95% confidence intervals (CI). *p*‐values are adjusted for multiplicity (false discovery rate). Models included random intercepts for subjects and fixed effects for Group, Time, and Group × Time interaction.

Abbreviations: ACIC + MF, autologous collagen‐induced chondrogenesis combined with microfracture; CI, confidence interval; FDR, false discovery rate; IKDC, International Knee Documentation Committee score; LMM, linear mixed‐effects model; Lysholm, Lysholm knee score; MF, microfracture; VAS, visual analogue scale for pain.

### 3.5 Structural Outcomes (MOCART)

**FIGURE 2:**
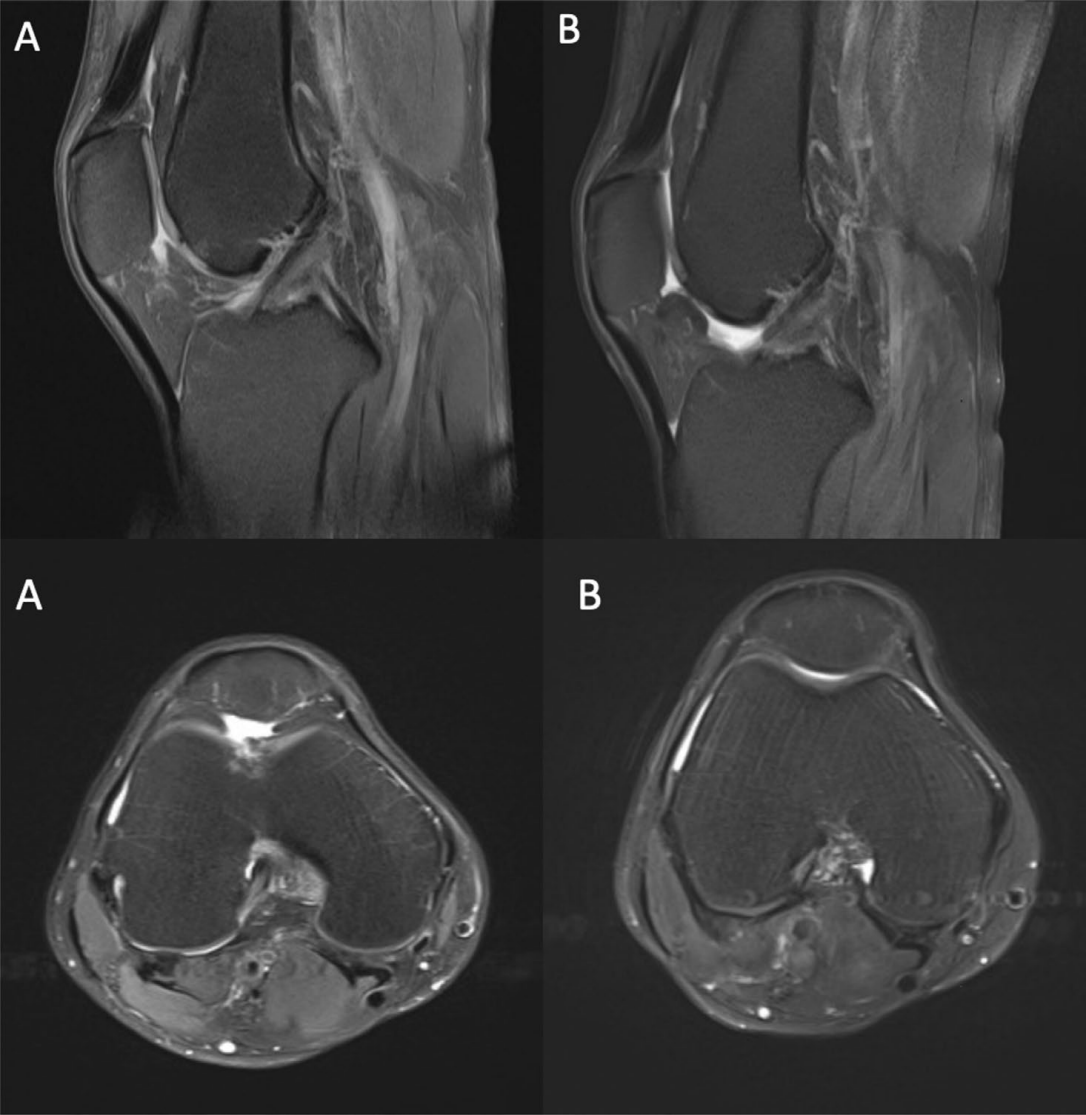
Representative MRI images of cartilage repair in the ACIC + MF group. Baseline and 5‐year postoperative scans are shown, illustrating defect filling, integration, and cartilage morphology.

**FIGURE 3:**
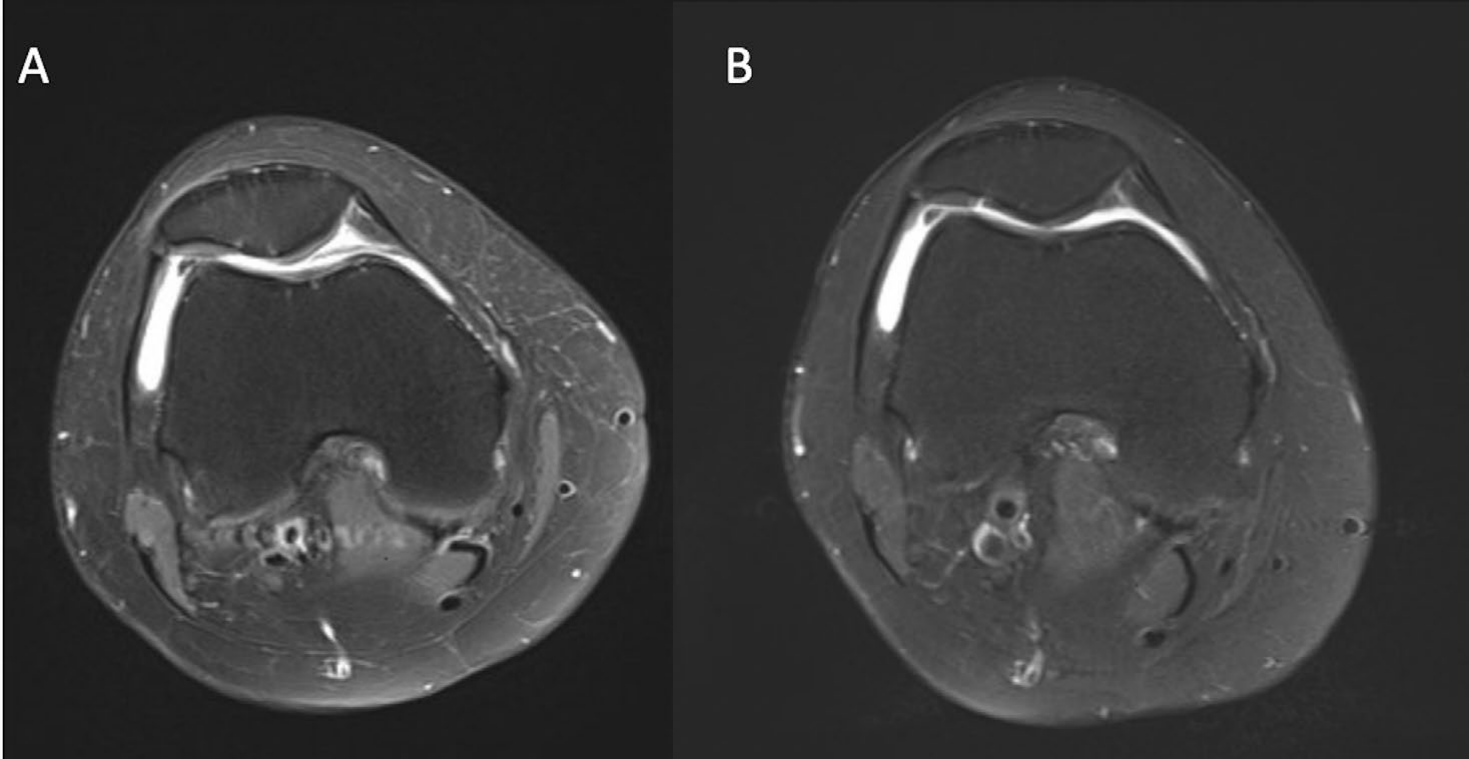
Representative MRI images of cartilage repair in the MF group. Baseline and 5‐year postoperative scans are shown for comparison.

Structural cartilage repair was assessed using MRI‐based MOCART scoring over the 5‐year follow‐up period. Representative MRI images illustrating cartilage repair morphology at baseline and at 5 years are shown for the ACIC + MF group and the MF group in Figures 2 and 3, respectively. In the ACIC + MF group, MRI demonstrated more complete defect filling, improved integration with adjacent native cartilage, and a more homogeneous repair tissue signal at long‐term follow‐up. In contrast, the MF group showed incomplete defect fill, irregular repair surfaces, and inferior integration in a substantial proportion of cases.

Quantitatively, MOCART scores increased over time in both groups; however, the magnitude and durability of improvement differed. Longitudinal analysis using linear mixed‐effects models revealed a significant Group × Time interaction at 5 years (*β* = 53.1, 95% CI 29.0–77.2, *p* < 0.001), indicating superior structural repair in the ACIC + MF group. At the 5‐year follow‐up, the mean MOCART score was 69.4 ± 17.9 in the ACIC + MF group compared with 23.6 ± 34.4 in the MF group. The bootstrap mean difference was approximately 46 points (95% CI 16.7–67.8), corresponding to a large effect size (Cohen's *d* = 1.32). Although the unadjusted Mann–Whitney *U* test yielded *p* = 0.0196, this difference did not remain statistically significant after multiplicity correction (adjusted *p* = 0.098; Table 6).

**TABLE 6:** MRI MOCART outcomes: Mean ± SD at each timepoint, within‐group changes (Δ ± SD), between‐group differences (bootstrap mean difference with 95% CI), Cohen's *d*, and FDR‐corrected *p*‐values.

Timepoint	MF mean ± SD	ACIC + MF mean ± SD	Δ vs. baseline (MF)	Δ vs. baseline (ACIC + MF)	Between‐group diff (95% CI)	Cohen's *d*	FDR‐adjusted *p*
Baseline	14.0 ± 12.2	9.5 ± 2.8			−4.4 (−12.5, 2.0)	−0.51	1.000
7 days	51.0 ± 20.0	46.5 ± 15.8	37.0 ± 15.1	37.0 ± 14.9	−4.5 (−19.5, 10.5)	−0.25	1.000
6 months	55.5 ± 19.9	64.0 ± 20.0	41.5 ± 17.2	54.5 ± 19.1	+8.5 (−7.5, 25.5)	+0.43	1.000
12 months	66.0 ± 26.1	83.0 ± 17.8	52.0 ± 29.7	73.5 ± 18.3	+16.9 (−1.5, 36.0)	+0.76	0.320
5 years	23.6 ± 34.4	69.4 ± 17.9	8.6 ± 41.4	60.0 ± 19.5	+46.0 (16.7, 67.6)	+1.75	0.098

*Note:* Values are mean ± standard deviation (SD). Δ indicates change from baseline. Between‐group differences are shown as bootstrap mean difference (95% confidence interval, CI). *p*‐values are Mann–Whitney tests with multiplicity correction using the false discovery rate (FDR). Cohen's *d* effect sizes were bootstrapped with 95% CI.

Abbreviations: ACIC + MF, autologous collagen‐induced chondrogenesis combined with microfracture; CI, confidence interval; FDR, false discovery rate; MF, microfracture; MOCART, magnetic resonance observation of cartilage repair tissue score; MRI, magnetic resonance imaging; SD, standard deviation.

**FIGURE 4:**
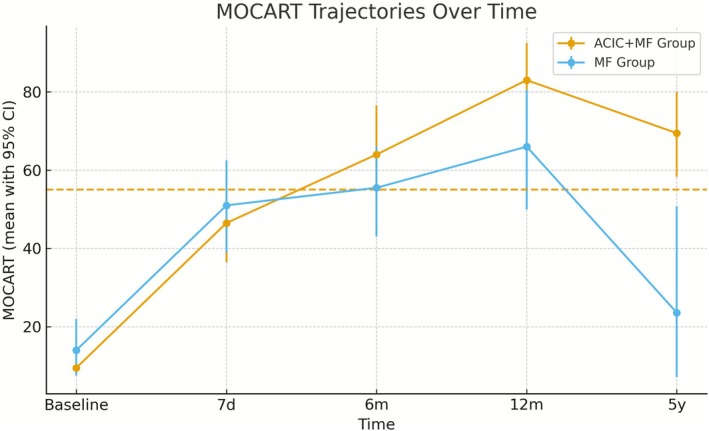
MOCART trajectories over time. Group trajectories of MOCART scores (mean ± 95% CI) at baseline, 7 days, 6 months, 12 months, and 5 years for ACIC + MF and MF. A dashed line at 55 denotes a literature‐based threshold associated with achieving PASS/KOOS‐QoL after cartilage repair (anchor‐based association; not an MCID for MOCART). Primary inference derives from the LMM Group × Time interaction at 5 years (*β* = 53.1, 95% CI 29.0–77.2, *p* < 0.001). Timepoint‐wise Mann–Whitney tests are descriptive and FDR‐adjusted; at 5 years, adjusted *p* = 0.098.

The longitudinal trajectories of MOCART scores for both groups across all follow‐up time points are illustrated in Figure 4, demonstrating early postoperative improvement in both groups and sustained long‐term structural superiority in the ACIC + MF group.

## 4 Discussion

### 4.1 Main Findings

In this 5‐year prospective randomized controlled trial, both ACIC combined with microfracture and microfracture alone resulted in significant improvements in pain and knee function; however, no between‐group superiority was detected in Lysholm, IKDC, or VAS scores. In contrast, ACIC + MF demonstrated a clear long‐term structural advantage, with significantly higher MOCART scores and a significant Group × Time interaction, indicating more robust morphologic cartilage repair.

### 4.2 Microfracture

Microfracture is a minimally invasive technique that was developed in the early 1980s by Steadman [10, 11, 12]. It uses an arthroscopic awl to drill small, equidistant holes in subchondral bone, at least 3–4 mm apart and 3–4 mm in depth, with 3–4 holes per 1 cm^2^ area. This procedure induces the migration of MSCs from the bone marrow to the cartilage defect to allow the formation of fibrocartilage. It was a good first‐line treatment for smaller lesions in patients aged < 45 years and the clinical results range from poor to good [13, 14]. Mithoefer et al. [15] conducted a systematic review about microfracture in 2009, it showed that the short‐term outcome was good, but the long‐term outcome was inconclusive, due to insufficient data. Shortcomings of microfracture included limited regeneration of hyaline‐like cartilage, variable volume of cartilage repair, and functional deterioration. Kruez et al. [16] reported that microfracture showed good short‐term results in small cartilage defects but poorer results at 18 months after surgery, as reflected by the Cincinnati Knee Rating System and International Cartilage Repair Society (ICRS) scores. This effect is clearer in large defects and in defects of the patellofemoral joint, where subchondral osteophytes develop in 20%–50% of cases. Gobbi et al. [17, 18] reported deterioration of scores in athletes 2–5 years after microfracture surgery, with the older ones together with multiple and large lesions having the worse prognosis. This might be explained in a meta‐analysis by DiBartola et al. [19], where the majority of the reparative tissue after microfracture was fibrocartilage and only 8.2% of patients yielded histological evidence of hyaline cartilage repair. Fibrocartilage, which is not as robust and durable as hyaline cartilage, has inferior wear characteristics and may lead to poorer clinical outcomes.

### 4.3 Autologous Collagen‐Induced Chondrogenesis

As a consequence, many cartilage regeneration techniques were designed to overcome these limitations of microfracture. More recently, a novel single‐stage surgical technique has been gaining popularity for cartilage regeneration. Autologous collagen‐induced chondrogenesis (ACIC) is a cost‐effective method and has no donor site morbidity for enhancing cartilage repair [20, 21, 22]. The ACIC technique is also called the Shetty–Kim technique because it was developed from the works of Shetty et al. [23]. Together, they developed the ACIC technique to be performed with one arthroscopic surgery, leaving wounds only for the arthroscopic portal incisions. In this procedure, microfracture combines with an exogenous scaffold to confine mesenchymal stem cells to the chondral defect and provide a conducive environment for them to differentiate into chondrocytes, forming new cartilage tissue and further repairing damaged cartilage. Even though ACIC is a relatively new technique, publishing limited literature showed excellent clinical results. Shetty et al. [24] were the first to describe the use of this kind of treatment in chondral defects in 2013. At 2 years after surgery, all patients had significant and sustained improvement in Lysholm score. Post‐operative MRI showed good cartilage defect filling with good MOCART scores. Kim et al. [25] compared porcine‐derived collagen‐augmented chondrogenesis to microfracture in 2020 in a multicenter, randomized control study. One hundred patients were randomly assigned to a microfracture or an investigational group. MOCART score, VAS, and KOOS pain score were significantly improved in the investigational group. In addition, the investigational group showed better filling of the cartilage defect in the knee joint. Silva et al. [26] compared ACIC to microfracture in 2020 based on clinical scores at 6 and 24 months. The ACIC group showed a significantly better SF36 mental function, IKDC and VAS at 24 months. Similarly, it also has been used for treatment of chondral lesions of the talus and results showed significant improvement in VAS and AOFAS scores at 6 months follow‐up.

### 4.4 Microfracture Combined With Autologous Collagen‐Induced Chondrogenesis

To our knowledge, there is a relative vacuum in the literature on the outcomes of such ACIC techniques, including comparison studies with performing microfracture alone. Our study aimed to compare clinical outcomes between the ACIC technique together with microfracture and microfracture alone over a 60‐month study period. Both groups showed improvements in Lysholm, IKDC, and VAS scores, and linear mixed‐effects models did not detect significant group differences or Group × Time interactions after multiplicity correction, indicating no functional superiority of ACIC + MF compared with MF. At 5 years, between‐group mean differences in clinical scales were small and fell well below published MCID thresholds, supporting the interpretation that any functional advantage was clinically negligible.

In contrast, structural assessment with MRI demonstrated a robust long‐term advantage for the ACIC + MF group, with significantly higher MOCART scores at 5 years, reflecting superior cartilage fill, integration with adjacent cartilage, and restoration of cartilage morphology. Although the unadjusted Mann–Whitney test at 5 years yielded *p* = 0.0196, this did not remain significant after conservative multiplicity correction (adjusted *p* = 0.098). Nevertheless, the bootstrap mean difference of approximately 46 points (95% CI 16.7–67.8) and the significant Group × Time effect in LMM (*β* = 53.1, 95% CI 29.0–77.2, *p* < 0.001) underscore a large structural effect favoring ACIC + MF.

Importantly, despite the structural advantage, functional outcome measures did not show between‐group superiority. At present, no universally accepted MCID has been defined for the MOCART score. Anchor‐based evidence suggests that an absolute threshold of approximately 55 points is associated with achieving PASS and KOOS‐QoL improvement in cartilage repair, although this does not constitute a formal change‐based MCID. In our cohort, the ACIC + MF group exceeded this clinically relevant threshold at 5 years, while the MF group did not, indicating that the structural difference may carry clinical significance despite absent functional superiority. These findings underscore the complex and imperfect correlation between morphologic repair and patient‐reported function and highlight the need for future studies to prospectively establish MOCART‐specific MCID and PASS thresholds.

Our findings should also be interpreted in light of prior pivotal studies. Kim et al. [25] reported that collagen‐based scaffolds combined with microfracture yielded significant improvements in KOOS pain and VAS scores at 24 months in a large multicenter RCT, whereas Shetty et al. [24] observed both Lysholm and MRI improvements in a smaller, uncontrolled series. In contrast, our 5‐year results revealed a clear structural advantage in MOCART scores but no demonstrable superiority in functional outcomes. This discordance may be explained by several factors. First, MRI‐based repair scores reflect morphological integration and fill, but they do not directly assess the biochemical quality of the tissue; fibrocartilage may appear structurally satisfactory yet lack the biomechanical durability of hyaline cartilage. Second, knee function is multifactorial, influenced by muscle strength, meniscal integrity, and alignment, which may attenuate the clinical impact of localized cartilage repair. Third, commonly used patient‐reported outcome measures such as Lysholm, IKDC, and VAS may lack the sensitivity to capture subtle improvements in daily function. Finally, our relatively small sample size limited statistical power to detect modest functional effects. Together, these considerations suggest that the benefits of the scaffold may be predominantly structural rather than functional within the 5‐year timeframe. Future studies integrating clinical, imaging, and—where possible—histological endpoints are needed to clarify the translational impact of structural cartilage repair.

### 4.5 Limitations and Prospect

The present study has some limitations. Foremost, the small sample size substantially limits statistical power. As a result, the trial should be viewed as an exploratory pilot study, and the absence of between‐group differences in functional outcomes may partly reflect insufficient power rather than true equivalence. In addition, the single‐center design may restrict generalizability, and although no patients were lost to clinical follow‐up, not all knees underwent MRI evaluation at 5 years, introducing the possibility of imaging‐related selection bias. The lack of histologic verification also precludes direct assessment of the biochemical quality of the repair tissue, and widely used PRO instruments such as Lysholm, IKDC, and VAS may lack the sensitivity to detect subtle benefits associated with focal cartilage regeneration. Future research should include adequately powered multicenter trials, integrate cartilage‐specific outcome measures and objective functional testing, and establish anchor‐based MCID and PASS thresholds for MOCART to better define the clinical significance of structural repair.

## 5 Conclusion

The combination of arthroscopic microfracture and collagen‐based scaffolds achieved promising long‐term structural repair in patients with knee cartilage defects, with significantly higher MOCART scores at 5 years compared to microfracture alone. However, these structural improvements did not translate into superior patient‐reported outcomes on Lysholm, IKDC, or VAS, which improved similarly in both groups. This structural–functional discrepancy highlights the complexity of cartilage repair evaluation and emphasizes that imaging outcomes alone may not fully capture patient‐important benefit. Future multicenter studies with larger samples and anchor‐based determination of MCID/PASS for MOCART are needed to clarify the clinical relevance of structural repair and to optimize patient‐centered treatment strategies.

## Author Contributions

**Shuofang Ren:** methodology, investigation, writing – original draft, writing – review and editing. **Zhuosong Bai:** investigation, writing – original draft, writing – review and editing. **Xin Lu:** investigation, visualization. **Yan Zhang:** formal analysis. **Xu Sun:** formal analysis. **Rui Tang:** formal analysis. **Bo Yang:** conceptualization, writing – review and editing. **Jun Qian:** methodology. **Guixing Qiu:** conceptualization, supervision.

## Funding

This research was supported by the National Natural Science Foundation of China CAMS Innovation Fund for Medical Sciences (2021‐I2M‐1‐052).

## Disclosure

We declare that all authors listed meet the authorship criteria according to the latest guidelines of the International Committee of Medical Journal Editors, and that all authors agree with the manuscript.

## Ethics Statement

This study involves human participants and was approved by the internal ethics review board of Peking Union Medical College Hospital (approval number: KYC‐IEC‐2022‐II‐05).

## Conflicts of Interest

The authors declare no conflicts of interest.

## Data Availability

The data that support the findings of this study are available from the corresponding author upon reasonable request.
